# Influence of Septal Deviation on the Prognosis of Transcanalicular Diode Laser-Assisted Dacryocystorhinostomy

**DOI:** 10.1155/2016/9573760

**Published:** 2016-04-06

**Authors:** Alberto Raposo, Francisco Piqueras, Francisco García-Purriños, María Ll. Martínez-Martinez, Jerónimo Lajara

**Affiliations:** ^1^University Hospital Morales Meseguer (UHMM), Calle Hazim de Cartagena, No. 38 Cartagena, 30204 Murcia, Spain; ^2^Department of Otolaryngology and Head and Neck Surgery, UHMM, Calle Hazim de Cartagena, No. 38 Cartagena, 30204 Murcia, Spain; ^3^Department of Otolaryngology and Head and Neck Surgery, UHAM, Murcia, Spain; ^4^Catholic University San Antoni, Murcia, Spain; ^5^Department of Ophthalmology, UHMM, Calle Hazim de Cartagena, No. 38 Cartagena, 30204 Murcia, Spain

## Abstract

*Purpose.* The objective of the present study is to determine whether the success rate in transcanalicular diode laser-assisted dacryocystorhinostomy (TCL DCR) is influenced by the variant septal deviation (SD).* Methods.* Patients were divided into two groups: one including operated lacrimal pathways (LP) with no anatomical nasosinusal variants and the other group of LP with SD. This study began on January 1, 2008, and ended on December 31, 2010, at Morales Meseguer Hospital. Variables were compared by means of ANOVA and a logistic regression model (LOGIT).* Results.* Out of the 159 LP operated on, 102 had no nasosinusal anatomic variant, but 39 LP were associated with SD. The first group evidenced a success rate of 67.64%, while the second group evidenced a success rate of 66.7%.* Conclusion.* We found no significant statistical differences between the success rates in the two groups (with SD and no anatomical variants). So we could avoid previous or concomitant septoplasty in some cases (mild and moderate SD).

## 1. Introduction

When a septal deviation (SD) is present during the endoscopy performed before a transcanalicular diode laser-assisted dacryocystorhinostomy (TCL DCR), one tends to think that this anatomical alteration could influence the final result, due to technical difficulties and alterations in osteotomy healing.

Dacryocystorhinostomy (DCR) is the surgical treatment for opening up lacrimal pathways [1, 2]. There are three types of DCRs: external, endoscopic, and transcanalicular approach [3–6].

We use the transcanalicular approach (TCL DCR) for all patients, except recurrences, which we treat externally.

Endoscopically nasosinusal anatomical variations may appear, such as septal deviation (SD), inferior turbinate hypertrophy, and concha bullosa [7–9]. SD seems to be the most frequent nasosinusal anatomic variant. Some patients have no symptoms in spite of SD [7].

We designed the present study in order to investigate the influence of nasal septal deviation on the surgery of lacrimal pathways. If significant influence cannot be proved, then previous or concomitant septoplasty could be avoided in candidates for surgery of the lacrimal pathways (LP).

## 2. Materials and Methods

From January 1, 2008, until December 31, 2010, one hundred and twenty-four patients were considered candidates for TCL DCR surgery, so they were included in this study.

A protocol was designed, including personal data, medical history, and degree of epiphora. In order to quantify and standardize the degree of epiphora more precisely, we used the Munk score [10]. Candidates for TCL DCR surgery had to meet the following conditions: need to dry tears more than 5 times a day (Munk score: 3–5), blockage of the vertical part of the lacrimal pathways, presence of lacrimal sac proved by dacryocystography, symptoms (epiphora), chronic dacryocystitis, and/or a history of acute episodes.

The patients were seen by the otorhinolaryngologist and the ophthalmologist in the same follow-up visit, in order to assess the feasibility of TCL DCR; septal deviations were noted. During this multidisciplinary consultation, the ENT specialist wrote down type and degree of SD. There are various classifications of SD [7, 11, 12]. We chose a classification based on the distance separating septum from lateral nasal wall [12]: mild (less than 50% the distance from septum to nasal wall); moderate (greater than 50% the said distance); and severe (septum touched the lateral nasal wall).

Patients younger than eighteen, with previous DCR (whatever the technique), with other disorders of ocular annexes, or lacking in motivation (they interfere with the Munk score) were excluded on this study.

All patients signed the consent form for TCL DCR to participate in this clinical trial and were operated on without taking into consideration the presence of SD or the lack of it.

This study was successfully approved by the ethics committee at Morales Meseguer Hospital.

A Varius laser was used during the surgery. This device uses an InGaAsP diode laser generator and a semiconductor with a wavelength of 980 nm (±5) with a maximum input power of 20 W. The corresponding silica optical fiber was sterile and disposable, measuring 600 microns. The ENT used a Karl Storz tube with an optical angle of 0 degrees.

During the postoperative period topical treatment was prescribed, with tobramycin and dexamethasone eye drops. Twenty-four hours after surgery nasal irrigation with saline water and topical nasal applications of fluticasone furoate were prescribed.

During the follow-up revisions endoscopies (one month, three months, and six months) were performed and traces of fibrin were removed; an assessment was also carried out, taking into account presence of epiphora (Munk scale), positive or negative nasal syringing with fluids, and endoscopic appearance of the osteotomy site.

Surgery was deemed “success” when the patients scored 0 or 1 on the Munk scale, in which they had to dry tears twice or less than twice a day, six months after the operation.

Therefore, we considered “failure” when they scored 2 to 5 on the Munk scale.

We divided the patients who had undergone TCL DCR into two groups: one group included LP with no anatomical nasosinusal variants and the other LP group was with SD or other nasosinusal alterations. Finally, we calculated the success rate for each group.

This prospective, nonexperimental clinical study was carried out correlating clinical features with a longitudinal analysis.

SPSS-20 software was used for estimation procedures.

Dichotomous variable “presence or absence of SD” has been studied and compared in both groups. Contrasts referred to equality of means of ANOVA like “duration of procedure,” “age,” and “duration of epiphora in years,” Pearson's correlation coefficient like “age” and “duration of epiphora,” or modeling explanatory ability of those predictor variables acting on probability of success, using the logistic regression model like “successful syringing at 3 and 6 months after operation,” “sex,” “bilaterality,” “right/left side,” “presence of granulomas,” “presence of synechiae,” “presence of postoperative granulomas,” and “presence of SD,” have been used.

## 3. Results

The study included 124 patients, in which 159 LP operations were performed, 102 LP (64.15%) did not have any anatomical variants, and 57 LP (35.84%) had anatomical variants. Out of these 57 LP with anatomical variants, 39 had SD (68.42%): in 21 of them it was mild (53.84%), in 15 it was moderate SD (38.46%), and in 3 it was severe SD (7.6%).

Other anatomical alterations were found in the remaining 18 LP (31.57%): one LP with concha bullosa (0%) and 17 LP with hypertrophic inferior turbinate (51%).

In the group with no anatomical alterations, 69 LP had a successful postoperative outcome (67.6%). In the SD group, 26 LP had a successful postoperative outcome (66.7%).

According to the LOGIT method, the difference between both groups in postoperative outcome was not significant (*p* > 0.05).

If we compare the success rate of the SD group (66.7%) and other anatomical alterations (44.1%) in our study, the difference is statistically significant (*p* < 0.05).

We also compared success rates in each SD group (mild, moderate, and severe).

The success rate for each group was as follows: 66.67% for mild SD; 66.60% for moderate SD; and 66.66% for severe SD.

In patients with severe SD (3), a previous septoplasty was necessary in order to make a correct TCL DCR possible later on, as a part of the same surgical procedure.

There were no complications during or after these procedures, no intraoperative bleeding, and no postoperative infection and all patients were discharged 4-5 hours after the operation.

## 4. Discussion

The demographic data collected for our sample agree with that found in worldwide medical literature, as far as sex [13–15], age [13–16], and race [15] are concerned.

There was a higher success rate in the SD group (66.7%) compared to the rest of anatomical alterations (success in this latter group amounted to 44.1%), but the former had a lower success rate when compared to the group with no anatomical alterations (67.6%).

The difference between those two groups was not statistically significant.

Most of septal deviations (69.6%) are inferior ridges or posterior deviations, so they did not need surgery. There is no need for previous or concomitant septoplasty in some cases like mild and moderate SD, because the success rate was similar.

All severe SD were operated on during the same procedure; therefore, access to the middle meatus in the nasal fossa presented no difficulties.

One month and three months later, most patients scored 0 to 1 on the Munk scale.

Stenosis, however, could recur due to fibrosis. It could change the scores from 0 to 2 over the course of the 6 months following surgery, so the success rate would decrease. After 6 months, the success rate remained stable.

We have not found articles referring to surgical success in patients with SD in scientific literature.

## 5. Conclusion

In our study, we found no significant statistical differences between the success rate in the two groups. Furthermore, we could avoid previous or concomitant septoplasty in some cases like mild and moderate SD because the success rate was similar to nonseptal deviation group. So we avoided risk of surgical complications like synechiae or granulomas.

## References

[1] Weil H. A. (1987). *Basic Dacryology. Diagnosis and Treatment*.

[2] Katowitz J. A., Hollsten D. A., Linberg J. V. (1988). *Lacrimal Surgery*.

[3] Adan J. C. (2006). *The Lacrimal System: Diagnosis, Management and Surgery*.

[4] Shun-Shin G. A., Thurairajan G. (1997). External dacryocystorhinostomy—an end of an era?. *British Journal of Ophthalmology*.

[5] Stammberger H., Posawetz W. (1990). Functional endoscopic sinus surgery. Concept, indications and results of the Messerklinger technique. *European Archives of Oto-Rhino-Laryngology*.

[6] Alañón F. (2008). *Estudio comparativo de la obstrucción del sistema nasolagrimal mediante DCR endocanalicular con Láser Diodo y DCR externa [Ph.D. thesis]*.

[7] Suárez C., Gil-Carcedo L. M., Marco J., Medina J., Ortega P., Trinidad J. (2007). *Tratado de Otorrinolaringología y Cirugía de Cabeza y Cuello*.

[8] Jamie S. (2004). The incidence of concha bullosa and its relationship to nasal septal deviation and paranasal sinus disease. *American Journal of Neuroradiology*.

[9] Hechl P. S., Setliff R. C., Tschabitscher M. (1997). *Endoscopic Anatomy of the Paranasal Sinuses*.

[10] Munk P. L., Lin D. T. C., Morris D. C. (1990). Epiphora: treatment by means of dacryocystoplasty with balloon dilation of the nasolacrimal drainage apparatus. *Radiology*.

[11] Guyuron B., Uzzo C. D., Scull H. (1999). A practical classification of septonasal deviation and an effective guide to septal surgery. *Plastic and Reconstructive Surgery*.

[12] Hong-Ryul J., Joo-Yun L., Woo-Jin J. (2007). New description method and classification system for septal deviation. *Journal of Rhinology*.

[13] Hong J. E., Hatton M. P., Leib M. L., Fay A. M. (2005). Endocanalicular laser dacryocystorhinostomy: analysis of 118 consecutive surgeries. *Ophthalmology*.

[14] Holzman P. (2009). Características clínicas y sociodemográficas de pacientes con epífora estudiada por dacriocistografía. *Archivos de Oftalmologia de Buenos Aires*.

[15] Roussos J., Bouzas A. (1973). Essai d’explication par des facteurs hormonaux de la grande fréquence d’apparition de la dacryocystite chronique chez les femmes plutôt que chez les hommes. *Société Française d'Ophtalmologie*.

[16] Schaudig U., Meyer-Rüsenberg H. (2009). Epiphora. Age-related changes of the ocular surface, eyelid function and the efferent tear ducts. *Ophthalmologe*.

